# [Expression of Concern] Effect of overexpression of HOX genes on its invasive tendency in cerebral glioma

**DOI:** 10.3892/ol.2026.15536

**Published:** 2026-03-18

**Authors:** Yun-Bao Guo, Yi-Meng Shao, Jing Chen, Song-Bai Xu, Xing-Dong Zhang, Mao-Ren Wang, Hai-Yan Liu

Oncol Lett 11: 75–80, 2026; DOI: 10.3892/ol.2015.3893

Following the publication of the above paper, it has been drawn to the Editor's attention by a concerned reader that, regarding the RT-polymerase chain reaction analyses shown in Fig. 3, the data shown for the *A9* and *D10 HOX* genes were strikingly similar; in addition, the data shown for the *B13* and *D13 HOX* genes were also remarkably similar. Furthermore, the blot for the *A9*/*D10 HOX* genes for the U-118 lane also looked very similar to the blot portrayed for the *D13 HOX* gene in both the U-138 and U-118 lanes, and the data shown for the *A6, A7, D4* and *D9 HOX* genes all bore a strikingly close similarity. Finally, it should be noted that a cursory inspection of the data shown for the RT-polymerase chain reaction analyses in Fig. 2 revealed similar potential anomalies with respect to strikingly similar looking data used to portray various of the *HOX* genes.

The authors have been contacted by the Editorial Office to offer an explanation for these apparent anomalies in the presentation of the data in this paper, and we are awaiting their response. Owing to the fact that the Editorial Office has been made aware of potential issues surrounding the scientific integrity of this paper, we are issuing an Expression of Concern to notify readers of these potential problems while the Editorial Office continues to investigate this matter further.

